# Efficiency and efficacy of planning and care on a post-anesthesia care unit: a retrospective cohort study

**DOI:** 10.1186/s12913-020-05376-2

**Published:** 2020-06-22

**Authors:** Bart van Tunen, Markus Klimek, Karin Leendertse-Verloop, Robert J. Stolker

**Affiliations:** Department of Anesthesiology, Erasmus MC, University Medical Center Rotterdam, P.O. Box 2040, 3000 CA Rotterdam, the Netherlands

**Keywords:** Anesthesia recovery period, Postoperative care, Mortality, Patient safety, Management of logistics, Queuing

## Abstract

**Background:**

In the post-anesthesia care unit in our hospital, selected postoperative patients receive care from anesthesiologists and nursing staff if these patients require intensive hemodynamic monitoring or treatment to stabilize vital functions (e.g., vasopressor use and mechanical ventilation support) during a one-night admission. We investigated the agreement between elective preoperative planning for post-anesthesia care unit admission and the postoperative reality, along with the consequences of planning failures.

**Methods:**

Data from records for 479 consecutive patients from June 1 to November 30, 2014, in a tertiary referral hospital were reviewed and analyzed. All patients admitted to PACU were included, along with patients scheduled to be referred to PACU but ultimately transferred to another ward. The primary outcome was the efficiency of planning PACU admission for elective patients. Secondary outcomes included secondary admissions to PACU or the intensive care unit (ICU) and 30-day morbidity and mortality.

**Results:**

Of the 479 included patients, 342 (71%) were admitted per preoperative planning. Five patients (1%) needed cardiopulmonary resuscitation, and six (1%) did not survive the follow-up period. Patients admitted to PACU because of a shortage of beds in the ICU had the highest readmission (20%) and mortality rates (20%) (*P* = 0.01).

**Conclusions:**

Preoperative planning for PACU admission was off-target for 29%. However, efficient care always takes precedence over efficient planning. In particular, downgrading patients to PACU because of a shortage of beds in the ICU was associated with a mortality increase.

## Background

In the post-anesthesia care unit (PACU) in our hospital, selected postoperative patients receive care from anesthesiologists and nursing staff if these patients require intensive hemodynamic monitoring or treatment to stabilize vital functions (e.g., vasopressor use and mechanical ventilation support) during a one-night admission [1]. The level of care in the PACU is lower than that provided in the ICU but higher than in other high-care units or common wards. PACUs have been established to improve OR logistics in times of chronically overloaded ICUs. In our hospital, preoperative planning for PACU admission is completely independent from logistics for the routine postoperative recovery ward and ICU planning.

Recent evidence suggests that both surgical procedure and the quality of postoperative care and complication management are major factors in patient outcomes [2, 3]. We view the availability of our PACU, as an opportunity to treat these patients in a way that enhances safety.

We hypothesized that preoperative planning of elective surgery according to our inclusion and exclusion criteria (Appendices 1a and 1b) would result in an optimal occupation of about 85% of our PACU beds, which is based on internal business plans and estimations according to the Queuing theory [4–6]. Our goal was to facilitate the maximum number of elective procedures while minimizing the cost of empty beds without risking a backlog of other elective surgeries.

## Methods

The study was approved by the Medical and Ethical Review Committee of our university medical center (number MEC-2016-014). The STROBE Statement checklist for cohort studies was followed in the writing of this manuscript.

The design of our PACU resulted from the need for a better perioperative flow for elective surgery. Our PACU consists of five beds; during our study period, a total of 562 PACU beds were available. In our perioperative planning, we work with capacity slots on PACU. These slots are made available for the different surgical disciplines according to a schedule, which is communicated about 6 weeks ahead of date. On our preoperative assessment policlinic, we plan our patients for postoperative admission location: general ward, PACU or ICU. On the day before, the OR planner and one of our staff anesthesiologists check all planned surgery and the postoperative admission location of all patients. According to these strictly applied planning rules, the number of patients planned for surgery on the same day with a documented indication for PACU admission may never be higher than the five beds we have. The selected patients, undergoing major but mostly uncomplicated interventions, as well as patients with particular comorbidities, are not admitted to an ICU. In a PACU, patients can receive a more intensive level of monitoring and care than in a general ward while the ICU burden can be relieved. However, in the ICU, a doctor is on the ward 24/7, whereas the medical care on specialist level in our PACU is provided on the ward from 8 a.m. to 8 p.m., with clinicians on call in-house during night. The nurse: patient ratio on our PACU is 1: 2 (same as on ICU) and paramedics such as a physiotherapist or dietist can be consultated during working hours from 9 a.m. to 5 p.m.

In this cohort study, all records for consecutive patients admitted to the PACU in our tertiary referral hospital between June 1 and November 30, 2014, were retrospectively reviewed. Cardiac and pulmonary surgeries were performed in our cardiothoracic surgery center, so these patients were not included in this study. All patients sent to the PACU were included, as were those who were scheduled to be admitted to the PACU but were placed on another ward.

Based on clinical experience, criteria for admission to PACU in our hospital consisted of anesthesiological indications, surgical indications, and/or comorbidities (Appendix 1a). Reasons for excluding patients are summarized in Appendix 1b. Elective surgery was defined as all planned surgery, as we could specifically study this elective surgery cohort, we excluded all emergency patients undergoing unplanned surgery within 24 h after hospital admission. Criteria for PACU discharge to the ward were a stable hemodynamic and respiratory status without vasopressor support and no need for invasive monitoring or treatment.

We classified included patients as follows: PACU planned/admitted (Group I); PACU planned/not admitted (Group II); and PACU not planned/admitted (Group III). For this third group, we subclassified patients further as upgraded to PACU because of anesthesiology- and/or surgery-related reasons/complications (Group III-a) or downgraded to PACU because of a full ICU (Group III-b).

The primary outcome of this study was the planning efficiency of elective patient admissions to PACU. Secondary outcomes were secondary admissions to PACU or ICU, cardiopulmonary resuscitation (CPR), and mortality. We also compared the mortality of patients admitted to the PACU with elective postoperative patients admitted to the ICU. We used a 30-day follow-up period, which is an established indicator of quality and safety of perioperative care [7].

### Statistical analysis

All data were analyzed using the Statistical Package for the Social Sciences (SPSS) version 22.0 (SPSS, Chicago, Ill., USA). Normality of continuous data was tested using the Shapiro–Wilk test. Continuous data are reported as means and standard deviation (parametric data) or as medians and percentiles (non-parametric data); categorical data are reported as numbers with percentages.

To compare outcomes between patients who were not planned for PACU but upgraded or downgraded to it, we used a contingency table and analyzed the results with Fisher’s exact test. Because of the scientific discussions about the need of multiple test adjustments for exploratory study design regarding secondary outcomes, we did not determine a specific level of significance [8].

## Results

**Fig. 1:**
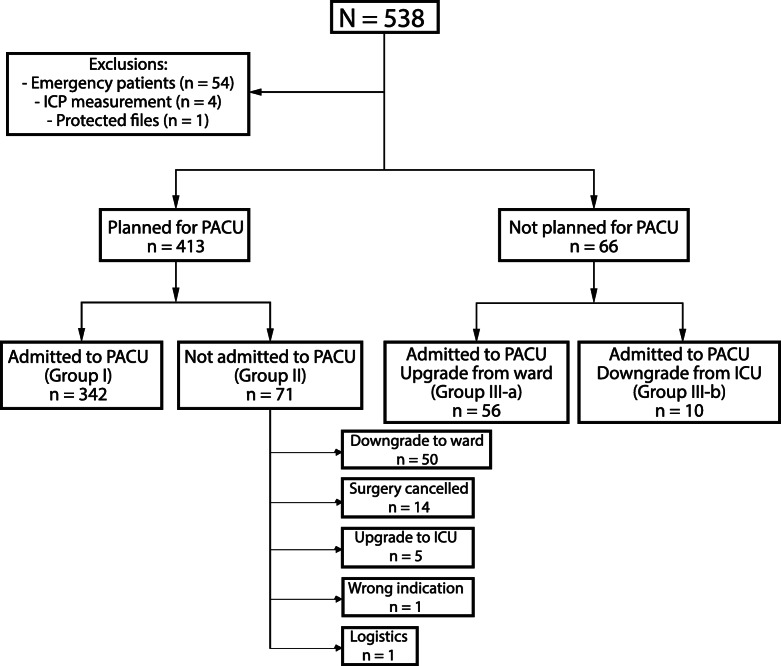
Overview of admission characteristics

From June 1 to November 30, a total of 538 patients (from a total volume of 4270 procedures performed) were admitted to the PACU or planned for admission. Of these, 59 patients were excluded from these analyses: 54 were emergency patients, 4 were admitted for measurement of intracranial pressure, and one patient had a protected electronic file, precluding chart review. Thus, we analyzed data for 479 patients (Fig. 1). Because of cancellation or delay of their procedure, 19 patients were included more than once in the database because they had more than one PACU indication. An overview of patient characteristics is shown in Table 1.

**Table 1:** Patient characteristics

**N**	479
**Sex (% of total cohort)**	
Men	280 (59%)
Women	199 (41%)
**Median age, years (P**_**25**_**–P**_**75**_**)**	61 (49–70)
**Mean height, cm (± SD)**	174 (±11)
**Median weight, kg (P**_**25**_**–P**_**75**_**)**	81 (70–94)
**Median BMI, kg.m**^**− 2**^**(P**_**25**_**–P**_**75**_**)**	26 (24–31)
**ASA**^**a**^**classification (% of total cohort)**	
I	48 (10%)
II	193 (40%)
III	218 (46%)
IV	20 (4%)

^a^American Society of Anesthesiologists

### Elective patients scheduled for PACU admission (groups I & II)

Of the 479 included patients, a total of 342 patients (71%) were planned for PACU admission and admitted to PACU (planned/admitted = Group I). Another 71 patients (15%) were preoperatively planned for PACU, but not admitted postoperatively (planned/not admitted = Group II), see Fig. 1.

An overview of the most common preoperative surgical and anesthesiological indications for a planned PACU admission according to our protocol is given in Table 2. Because some patients had more than one indication, 498 indications for PACU admission existed for the 413 patients who were planned preoperatively (Group I + II). Of these 413 patients, the indication for 184 patients (45%) was anesthesiological, comorbidity, or other reason (Table 2, b–d) rather than type of surgery. We also counted 43 other indications that are not mentioned in Appendix 1a (e.g., minor/moderate surgery in frail patients and rare, unlisted major high-risk procedures). Frail patients were considered as high risk and were planned for PACU admission. Frailty was defined individually by the screening anesthesiologist in the preoperative outpatient department, based on declines in physiologic reserve and function across multi-organ systems, leading to increased vulnerability for adverse health outcomes [9]. In a subgroup analysis of these 43 patients, we could not identify any specific listed risk factor.

**Table 2:** Elective patients scheduled for PACU admission

Preoperative admission indication	Indications, total cohort (***N*** = 413)	Indications, planned and admitted patients (Group I) (***n*** = 342)	Indications, planned but not admitted patients (Group II) (***n*** = 71)
**a. Surgical indications**^a^**(*n*** **= 229)**	**229**	**204**	**25**
Supratentorial craniotomy (including open biopsy)	89	82	7
Whipple operation	29	25	4
Hemihepatectomy	26	20	6
Kidney transplantation + comorbidity or need for vasopressive support	16	15	1
Awake craniotomy	11	10	1
**b. Anesthesiological indications (*n*** **= 47)**	**55**	**38**	**17**
(Expected) perioperative pulmonary or cardiac complications	44	30	14
(Expected) postoperative catecholamine support	6	5	1
(Expected) postoperative airway complications	5	3	2
**c. Comorbidities (*n*** **= 99)**	**171**	**137**	**34**
OSA syndrome	70	54	16
Heart failure	67	54	13
Chronic obstructive pulmonary disease	17	14	3
Morbid obesity	14	12	2
Cervical paraplegia	2	2	0
Unregulated diabetes mellitus	1	1	0
**d. Other (not listed in** Table 1**) (*n*** **= 38)**	**43**	**34**	**9**
Minor surgery in frail patients	17	14	3
Rare major high-risk surgery	18	13	5
Congenital syndromes	5	4	1
Bronchoalveolar lavage	3	3	0
**Total (*n*** **= 413)**	**498**	**413**	**85**

^a^ Most relevant surgical indications

The biggest contributor to downgrading in the PACU planned/not admitted group (Group II) was obstructive sleep apnea (OSA) syndrome as the preoperative indication. Most patients were not admitted because the attending anesthesiologist assessed the early postoperative condition as better than expected and agreed with admission to the general ward.

### Elective patients not scheduled for PACU admission, but subsequently admitted to PACU (group III)

Of the 137 patients (29%) that were not planned correctly, 66 patients (14%) were not scheduled preoperatively for PACU admission but were admitted (not planned/admitted = Group III), see Fig. 1. Fourteen of these underwent surgery associated with one of the PACU indications listed in Appendix 1a and should have been scheduled preoperatively to conform to protocol. Focusing on the anesthesiological reasons, two patients diagnosed with cardiac comorbidities underwent kidney transplantation, one patient with cardiac arrhythmias and decompensation underwent carotid surgery, and the other patient underwent cervical spondylodesis.

Forty-two patients who were not planned for PACU but upgraded to it from the general ward underwent heterogeneous types of operations showing perioperative complications, such as suspect changes in electrocardiography or arrhythmia (*n* = 7), need for catecholamine support because of prolonged intraoperative hypotension (*n* = 6), metabolic acidosis and electrolyte disorders (*n* = 5), pulsoximetric saturation drops (*n* = 3), and massive perioperative blood loss (*n* = 1).

Ten patients were planned for ICU admission but were downgraded to PACU immediately postoperative because of unavailable ICU beds. In these cases, at surgery start, a postoperative bed was available in ICU but was occupied by an emergency patient during the procedure. With agreement from both the attending anesthesiologist and surgeon, in these cases, the patient planned for ICU was not transferred to another hospital but instead was admitted to PACU with back-up support of the intensivist, if necessary.

### Follow-up

Of 413 patients scheduled for admission to PACU, 28 (7%) were secondarily admitted to ICU or readmitted to PACU within 30 days after the initial postoperative admission (Table 3), and another three were admitted a third time within that 30-day period. Secondary and tertiary admissions were mainly because of cardiovascular (*n* = 10) or surgical reasons (*n* = 7), such as bleeding or ileus. Other reasons were pulmonary (*n* = 5), neurologic (*n* = 5), infectious (*n* = 5), or metabolic (*n* = 1). Of the three patients admitted a third time, two had developed postoperative complications and underwent another surgery, followed by a second admission to PACU. The third patient was readmitted for complex pain treatment.

**Table 3:** Secondary and tertiary PACU and ICU admissions

Readmission	Total cohort (***N*** = 479)	PACU: planned/admitted (Group I) (***n*** = 342)	PACU: planned/not admitted (Group II) (***n*** = 71)	PACU: not planned/admitted instead of general ward (Group III-a) (***n*** = 56)	PACU: not planned/admitted instead of ICU (Group III-b) (***n*** = 10)
**Secondary admission**	**28**	**19**	**3**	**4**	**2**
PACU	3	2	0	1	0
ICU	25	17	3	3	2
**Tertiary admission**	**3**	**3**	**0**	**0**	**0**
PACU	1	1	0	0	0
ICU	2	2	0	0	0
**Total (% of total group number)**	**31 (6%)**	**22 (6%)**	**3 (4%)**	**4 (7%)**	**2 (20%)**

Subgroup analysis showed that the patients who were admitted to PACU because of unavailable beds in the ICU (group III-b) had the highest readmission rate, at 20% (Table 3).

Of all 479 inclusions, five patients (1%) underwent CPR during the 30-day follow-up period after surgery, two of them unplanned transfers to PACU. Another six patients (1%) did not survive this period (Table 4), two of them among the patients who were downgraded from ICU, for a rate of 20% (2/10) in this group. This mortality rate in group III-b was higher than among patients who were planned for and admitted to PACU (group I, 4/342 (1%), *P* = 0.01), planned for and not admitted to PACU (group II, 0/71 (0%), P = 0.01), and not planned for but upgraded to PACU (group III-a, 0/56 (0%), *P* = 0.02).

**Table 4:** CPR and mortality

Outcome	Total cohort (***N*** = 479)	PACU: planned/admitted (Group I) (***n*** = 342)	PACU: planned/not admitted (Group II) (***n*** = 71)	PACU: not planned/admitted instead of general ward (Group III-a) (***n*** = 56)	PACU: not planned/admitted instead of ICU (Group III-b) (***n*** = 10)
CPR	5	3	0	2	0
Death	6	4	0	0	2
**Total (% of total group number)**	**11 (2%)**	**7 (2%)**	**0 (0%)**	**2 (4%)**	**2 (20%)**

## Discussion

In this study, we analyzed data for 479 patients with a preoperative indication for admission to PACU or with an unplanned PACU admission. Of these, 137 patients (29%) had not been planned correctly. The proportion of these incorrect planned patients is roughly unchanged throughout the study period (Appendix 2). Most of these cases of unplanned PACU admission would have been unforeseen, but 14 of them could have been planned correctly if our guidelines had been followed.

Although the primary outcome of efficiency of planning for PACU admission was only 71%, secondary outcomes (readmissions, CPR, and mortality) were relatively low in the group planned for PACU but not admitted. These results suggest that the decision to place these patients on the general ward instead was appropriate. However, for each of these 71 patients planned for PACU but not admitted, a dispensable bed was reserved and another elective surgery might not have been planned that day. Most patients in this group were indicated for PACU because of OSA, which is considered a legitimate reason for PACU admission because of the increased risks for postoperative complications [10]. Multiple studies highlight the fact that most respiratory complications from OSA syndrome manifest in the first 24 h postoperatively [11, 12], suggesting that its inclusion on the PACU indications list is appropriate.

Seventeen patients considered to be frail were planned for and admitted to PACU without an indication on the prespecified list of indications (Appendix 1a). Admission indications should be revised with the inclusion of a clear definition of frailty, using a validated scoring system such as the Risk Analysis Index, the Hopkins Frailty Score, or Risk Stratification Indices [13–15].

Readmission is associated with adverse health events during the same hospitalization, and mortality rates are up to 11 times higher in these patients than in those who are not readmitted [16–19]. Furthermore, postoperative complications have been identified as a major contributor to short- and long-term mortality, and these patients are at higher risk of intervention from an emergency team [20–22]. Among patients not planned for PACU but admitted, the high number of readmissions to PACU and secondary/tertiary admissions to ICU, together with the highest mortality rate among those shifted to PACU from ICU, stresses the need to admit patients to the right unit. However, according to Warner et al., more than one third of major morbidity occurs 48 h or more after surgery [23], making a zero readmission rate quite difficult to achieve.

Regarding multiple testing for our secondary outcomes, discussion exists for adjusting the level of significance in exploratory study design. Therefore we did not determine a strict level of significance in our methods. However, no matter what level of significance is used, we found an increased mortality rate in the patients shifted from ICU to PACU (group III-b) and there is a clear trend that the difference between these patients and the patients from groups I (*P* = 0.01), II (*P* = 0.01) and III-a (*P* = 0.02) can be considered significant. During the same 6-month period, 222 elective postoperative (non-cardiac surgery) patients were admitted to ICU. Of this group, six (3%) died. When we compare the mortality of these patients with those in the current study who were shifted from ICU to PACU, mortality was higher in the latter group (*P* = 0.04). The shift of a patient from ICU to PACU means reduced clinician presence during the nighttime hours, making such a shift potentially riskier for the patient. This risk should be considered in making such decisions. Our results suggest that moving patients planned for ICU to PACU is highly undesirable, but the retrospective nature of this study precludes further conclusions or recommendations.

Our PACU differs in important ways from those in some other countries. As mentioned the introduction, our PACU provides intensive hemodynamic monitoring and treatment to stabilize vital functions during a one-night admission for selected patients undergoing high-risk surgery or who have high-risk comorbidity. This mission differs significantly from a phase I PACU (which in our hospital is known as the recovery ward), where any postoperative patient recovers from surgery until they are discharged to an alternative location (e.g., a phase II discharge unit) the same day [24]. With this clarification, it remains of utmost importance to admit the right patient to the right unit and to prevent logistical issues (such as unavailable ICU beds) that require large changes to organizational processes.

Only three other studies have focused on this specific subject and are available for comparison. Irone et al. included 1142 patients admitted to a comparable PACU [25]. In their study, hospital mortality was much higher (5.6%) than in our patient group, and they faced ICU-related logistic issues for 26% of their patients (compared to 0.02% or 10/479 in our study). Their findings, however, are in agreement with ours in that it is a challenge for intensive care medicine to identify with sufficient lead time which patients are vulnerable.

Bing-Hua found that prolonged waiting (≥6 h) in a recovery ward such as one type of PACU is associated with higher ICU mortality [26]. These results also are in agreement with ours. This apparently common phenomenon of a shortage of ICU beds highlights the importance of reliable preoperative planning.

According to Wickboldt et al., the duration of PACU stay is an independent risk factor for mortality and might be a surrogate marker for early postoperative adverse events or complications [27]. In our study, eight patients stayed for more than 1 day in the PACU. None of them were readmitted or underwent CPR, but one patient died 3 days after surgery because of a major cerebrovascular infarction.

### Limitations

We want to highlight a few limitations of our study, the most relevant of which are related to its retrospective design. For logistical reasons, a sample size calculation was not possible because of a lack of international literature, so we decided that a 6-month retrospective design would be representative and sufficient. When our study was registered at our hospital’s ethical committee in 2016, the study period from June to November 2014 was considered as most representative with regards to available beds, number of admissions and the nurse: patient ratio. Furthermore, in contrast to later periods, during these 6 months no major changes in the processes were implemented (e.g. later we had implementation of a new electronic patient-data-system, renovation and even moving of the ward). So, whilst these data might seem old, they still reflect our current way of planning and admitting patients to our PACU and we are convinced that the results are easy transferable to today’s situation.

Furthermore, this is a single-center study in a university hospital based on our local policy and practices, which cannot easily be transferred to other hospital settings, and the small number of included patients precludes strong conclusions. However, we believe that the results presented here can be useful for colleagues working in different settings, as well.

Despite these limitations, this study highlights the need for reliable planning of the postoperative admission location and the effect of any deviation from this planning. It is important to recognize that a well-considered deviation of the planned admission location – although it might seem to limit planning efficiency – supports efficient, safe, patient-directed and goal-directed high-quality care, as described by the World Health Organization, even if this results in an empty bed on the PACU [28].

## Conclusion

The appropriate postoperative destination of almost 29% of all patients included in this study had not been planned correctly. This level of inefficiency traces to incorrect preoperative assessment by the surgeon or anesthesiologist, an incomplete list of PACU indications, logistic issues related to ICU bed availability, and changes in the perioperative course. However, it is important to emphasize that efficient care always takes precedence over efficient planning. Downgrading a postoperative patient from PACU to general ward based on decision by the anesthesiologist in charge creates no additional risk for the patient and enhances efficiency of care. On the other hand, despite the small number of patients, we can conclude that there is a clear trend (*P* = 0.01) that an unplanned shift from ICU to PACU seems to be associated with an increase in mortality risk.

### Implications

The findings presented here can be used by others to review (and hopefully improve) their own practice concerning the perioperative patient flow. This begins with the identification and selection of patients needing a more intense level of postoperative care than a general ward can provide, but includes also the optimization of the logistics around these beds providing the higher level of care. Our findings highlight the fact that patients downgraded from ICU to PACU are vulnerable and prone for complications and mortality. This situation should be prevented whenever possible, and otherwise deserves special attention from the responsible physicians. However, shortage of beds in ICUs is an increasing common phenomenon worldwide and management of these logistic issues and its impact on the quality of care should be addressed by future research.

## Supplementary information

**Additional file 1.** Appendices.

## Data Availability

The datasets used and/or analysed during the current study are available from the corresponding author on reasonable request.

## Abbreviations

PACU: Post Anesthesia Care Unit
ICU: Intensive Care Unit
CPR: Cardiopulmonary resuscitation
OSA: Obstructive sleep apnea
OR: Operation Room
