# Supplementary material for: Efficiency and efficacy of planning and care on a post-anesthesia care unit: a retrospective cohort study
Source: BMC Health Serv Res. 2020 Jun 22;20:566. doi: 10.1186/s12913-020-05376-2 (PMC7310230; doi:10.1186/s12913-020-05376-2)
Supplement: Supplementary file 1 — Additional file 1. Appendices. [file 12913_2020_5376_MOESM1_ESM.docx]

**Appendices**

Appendix 1a. Inclusion criteria

| **Anesthesiological indications**  (Expected) postoperative airway complications  (Expected) perioperative pulmonary or cardiac complications  (Expected) postoperative catecholamine support | **Comorbidities**  Cervical paraplegia  COPD  Heart failure  Morbid obesity  Neuromuscular disease  OSA syndrome  Unregulated diabetes mellitus |
| --- | --- |
| **Surgical indications**  All maxillofacial surgery with difficult postoperative airway management  Awake craniotomy  Axillofemoral bypass  Carcinoid tumor + carcinoid related comorbidity  Carotid surgery or stenting + comorbidity  Cervical or high-thoracal laminectomy  Craniofacial surgery  Cystoprostatectomy  Debulking ovarian cancer  Elective coiling of cerebral aneurysm  Free flap face or head  Free flap or mamma reconstruction + comorbidity  Glomus tumor  Hemihepatectomy  Hemipelvectomy  Intrathecal baclofen pump + severe comorbidity  Kidney transplantation + comorbidity or need for vasopressive support  Maxillofacial surgery with opening dura  Microvascular decompression of trigeminal nerve  Minor craniofacial surgery | Minor elective clipping of cerebral aneurysm  Minor intracranial arteriovenous malformation  Minor surgery to posterior fossa (including vestibular schwannoma)  Neck dissection with free flap  Posttraumatic or major dorsal spondylodesis  Radical prostatectomy with lymphadenectomy  Radical Wertheim hysterectomy  Revision of total hip replacement or Girdlestone’s operation  Stenting of aorta (acute)  Supratentorial craniotomy (including open biopsy)  Thymectomy  Tips procedure  Total larynx extirpation  Pheochromocytoma  Radiofrequency ablation in cirrhotic liver  Thyroidectomy with sternotomy  Total gastrectomy  Transsphenoidal resection of pituitary gland + comorbidity  Whipple procedure |

Appendix 1b. Exclusion criteria

| **Exclusion criteria (before admission)** |
| --- |
| Patients after resuscitation, unless capacity constraints on Intensive Care Unit |
| Patients suffering a subarachnoid hemorrhage |
| Preoperative admissions for liver transplantation |
| Patients after lung transplantation |
| Intoxications admitted by emergency department |
| Patients requiring isolated nursing |
| Patients expecting to need more than 24 hours of intensive care and monitoring |

Appendix 2a. Proportion of incorrect planned patients throughout study period (table)

| **Week** | **Number of correct planned patients (Group I)** | **Number of incorrect planned patients**  **(Group II & III)** | **Proportion of incorrect planned patients** |
| --- | --- | --- | --- |
| **1-2** | 32 | 9 | 22% |
| **3-4** | 23 | 13 | 36% |
| **5-6** | 26 | 10 | 28% |
| **7-8** | 30 | 5 | 14% |
| **9-10** | 24 | 17 | 41% |
| **11-12** | 11 | 8 | 42% |
| **13-14** | 24 | 12 | 33% |
| **15-16** | 29 | 9 | 24% |
| **17-18** | 32 | 10 | 24% |
| **19-20** | 30 | 14 | 32% |
| **21-22** | 20 | 10 | 33% |
| **23-24** | 32 | 10 | 24% |
| **25-26** | 29 | 10 | 26% |
|  |  |  |  |
| **Mean ± SD** |  |  | **29% ± 8%** |
| **Total (% of total cohort)** | **342 (71%)** | **137 (29%)** |  |

Appendix 2b. Proportion of incorrect planned patients throughout study period (figure)
